# Prevalence and Correlates of Vitamin D Deficiency and Insufficiency in Luxembourg Adults: Evidence from the Observation of Cardiovascular Risk Factors (ORISCAV-LUX) Study

**DOI:** 10.3390/nu7085308

**Published:** 2015-08-13

**Authors:** Ala’a Alkerwi, Nicolas Sauvageot, Georges Gilson, Saverio Stranges

**Affiliations:** 1 Luxembourg Institute of Health (LIH) (formerly the Centre de Recherche Public Santé), Centre d’Etudes en Santé, Grand-Duchy of Luxembourg, L-1445 Strassen, Luxembourg; E-Mails: nicolas.sauvageot@lih.lu (N.S.); saverio.stranges@lih.lu (S.S.); 2 Department of Clinical Biology, Centre Hospitalier du Luxembourg, L-1210 Luxembourg, Luxembourg; E-Mail: Gilson.Georges@chl.lu

**Keywords:** vitamin D deficiency, cross-sectional, population-based study

## Abstract

Evidence on vitamin D status and related risk factors in Luxembourg adults is lacking. This study aimed to determine the prevalence of vitamin D deficiency and insufficiency and related risk factors among healthy adults in Luxembourg. Based on clinicians’ observations, it was hypothesized that vitamin D deficiency and insufficiency might be common in our population, constituting a significant public health concern. A nationally representative random sample of 1432 adults was enrolled in the ORISCAV-LUX study, 2007–2008. The participants were divided into four categories according to their serum concentrations of 25-hydroxyvitamin D [25(OH)D]. Descriptive, univariate and multivariate statistical analyses used weighted methods to account for the stratified sampling scheme. Only 17.1% of the population had a “desirable” serum 25(OH)D level ≥75 nmol/L, whereas 27.1% had “inadequate” [serum 25(OH)D level 50–74 nmol/L], 40.4% had “insufficient” [serum 25(OH)D level 25–49 nmol/L], and 15.5% had “deficient” [serum 25(OH)D level <25 nmol/L)]. The prevalence of vitamin D deficiency was greater among current smokers, obese subjects, those having reduced HDL-cholesterol level and fair/poor self-perception of health, compared to their counterparts. The prevalence of vitamin D insufficiency was additionally higher among nondrinkers of alcohol, Portuguese and subjects from non-European countries. The final multivariate logistic regression analyses revealed that smoking status and obesity were independent correlates of vitamin D deficiency and insufficiency, respectively. Inadequate vitamin D status is highly prevalent among adults in Luxembourg and is associated with specific lifestyle factors. Along with the effect of vitamin D deficiency and insufficiency on the risk of several diseases, cancer and mortality, our findings have practical implications for public health dietary recommendations, and of particular importance for healthcare practitioners and policy makers.

## 1. Introduction

During the last decade, there has been a growing interest in the beneficial effects of vitamin D on a wide range of health outcomes. It is well-recognized that this nutrient plays an important role in bone mineralization and other metabolic processes in the human body such as calcium (Ca) and phosphate homeostasis and skeletal growth [1].

Serum 25-hydroxyvitamin D [25(OH)D] concentration is the parameter of choice for the assessment of vitamin D status; as it reflects vitamin D exposure, incorporating endogenous synthesis from solar exposure, dietary intake from foods, fortified products, and/or supplements [2]. Although there are some areas of debate in relation to vitamin D requirements and the impact of vitamin D intake and status on many aspects of human health, there is a general agreement that prevention of vitamin D deficiency and insufficiency should be considered as a public health priority [3]. Given its key role in skeletal health, adequate vitamin D status has important implications in bone loss, muscle weakness and falls and fragility fractures in older people. These are highly significant public health issues in terms of morbidity, quality of life and costs to health services.

In addition, a plethora of recent publications emphasized that vitamin D possibly plays a much broader role than simple beneficial effects on skeletal tissues. Latest evidence has suggested that low serum concentrations of 25(OH)D are associated with a number of non-skeletal disorders including cancer [4,5], infections [6,7], auto-immune diseases [8] and cardiovascular disease [9,10]. Vitamin D supplementation might mitigate the incidence of these diseases and reduce all-cause mortality [11,12].

Vitamin D deficiency and insufficiency has been documented as a frequent public health problem in Europe and worldwide with striking geographical variations [3,13,14,15]. At a global level, an estimated 1 billion people have inadequate levels of vitamin D in their blood, across all ethnicities and age groups [15,16,17]. In recent years, there have been several reports suggesting a high prevalence of low vitamin D intakes and an inadequate vitamin D status in Europe [3].

Luxembourg is one of the few European countries without epidemiological evidence on the prevalence of vitamin D status and deficiency among the general adult population, however accurate assessment of the distribution of vitamin D status may help decision-makers to develop coherent and effective strategies for the prevention and treatment of inadequate vitamin D status.

Therefore, the aim of this study was to assess vitamin D status and prevalence of vitamin D deficiency and insufficiency in a national cohort of adults aged 18 to 69 years who participated in the ORISCAV-LUX study in 2007–2008. An additional aim was to examine determinants of vitamin D deficiency and insufficiency and compare prevalence estimates by demographic, socioeconomic factors and health conditions within this population. Based on clinicians’ observations, it was hypothesized that vitamin D deficiency and insufficiency might be common in Luxembourg’s population, thus constituting a significant public health concern.

## 2. Material and Methods

### 2.1. Study Design and Participants

The ORISCAV-LUX study was a nationwide population-based survey conducted in 2007–2008 to determine the prevalence of potentially modifiable cardiovascular disease risk factors in adult population resident in Luxembourg. A detailed description of the methodology has been described elsewhere [18,19]. Briefly, a representative sample of the national population was drawn from the national health insurance registry, stratified by gender (male and female), age (5-year categories) and districts of residence (Luxembourg, Diekirch and Grevenmacher). A total of 1432 non-institutionalized adults aged 18 to 69 years were successfully enrolled. Of these, blood specimens for analysis of serum 25(OH)D were available for 1352 people. However, the final sample available for analyses was made by 1335 subjects, after excluding 17 subjects taking vitamin supplements and missing data for body mass index (BMI) (1 subject), HDL-C (29 subjects) and self-perceived health (32 subjects).

### 2.2. Demographic Variables

Demographic details including age, sex, country of birth, economic status and educational attainment, were collected by trained research nurses using standardized questionnaires. These variables were categorized as follows: sex: men, women; age: 18–29 years, 30–49 years, 50–69 years; country of birth: Luxembourgish, Portuguese, other European, non-European; education: primary, secondary, tertiary level; income: living above, below poverty threshold. Further details regarding the collection of information on these variables have been published elsewhere [20].

### 2.3. Lifestyle-Related Variables

Physical activity during the last 7 days before the interview was assessed using the International Physical Activity Questionnaire (IPAQ). The studied population was categorized into physically inactive and active [21].

Height, weight and waist circumference (WC) were measured using standard procedures. BMI was calculated as weight in kg divided by height in m^2^. Participants with BMI below 18.5; 18.5–24.9; 25.0–29.9; ≥30 kg/m^2^ were considered as “underweight”; “normal weight”; “overweight” and “obese” subjects respectively. Abdominal obesity was defined as WC ≥ 102 cm for men and WC ≥ 88 cm for women [22]. Smoking status was categorized into “current smoker” *vs.* “ex- and never-smoker”. High HDL cholesterol was defined as ≥40 mg/dL for men and ≥50 mg/dL in women [23].

The participants were asked to self-report their health as excellent, good, fair and poor. This variable was regrouped into: Excellent/good *vs.* fair/poor. Dietary intake data were collected using a validated self-administered semi-quantitative food frequency questionnaire (FFQ) [24], including dairy intake in servings/day.

### 2.4. Vitamin D Measures

Blood was drawn from participants after an overnight fast (minimum 8 h), centrifuged and transported daily to the central laboratory. All the analyses for 25(OH)D were performed in the same laboratory using a COBAS e601 line with an Electro-Chemi-Luminescent-Immuno-Assay (ECLIA) kit (Roche Diagnostics, Rotkreuz, Switzerland). The measuring range extended from 7.5 to 175 nmol/L and functional sensitivity was at 10.0 µg/mL. In this laboratory, between-run precision at concentrations of 51.6 and 100 nmol/L was 6.2% and 5.7%, respectively.

### 2.5. Definition of Vitamin D Status

For international comparative purposes, we used cut-offs suggested by the International Osteoporosis Foundation to define different categories for vitamin D status. These levels were used to develop a worldwide map providing a global representation of vitamin D status [25,26]: In decreasing order of severity, the serum 25(OH) D levels were as follows: Vitamin D deficient, <25 nmol/L; vitamin D insufficient, 25–49 nmol/L; vitamin D inadequate, 50–74 nmol/L and desirable level, ≥75 nmol/L.

These thresholds are in line with a recent overview of vitamin D status in Europe (3), which considered a blood 25(OH)D concentration below 25 nmol/L as the lower threshold of vitamin D status and/or an indicator of risk of vitamin D deficiency. From prevention and public health standpoint, it is also interesting to investigate the group of people at risk of having insufficient level of vitamin D (25(OH) D level <50 nmol/L), since this status may concern larger segments of the population.

### 2.6. Ethical Aspects

The present study was conducted according to the guidelines laid down in the Declaration of Helsinki, and all procedures involving human subjects were approved by the National Research Ethics Committee (N 200609/03) and the National Commission for Private Data Protection. Written informed consent was obtained from all the participants.

### 2.7. Statistical Analysis

Proportion of serum 25(OH)D levels <25, 25–49, 50–74 and ≥75 nmol/L were computed and presented as a bar chart. Means (±SE) of serum (OH) D levels by age, sex and country of birth were calculated. To account for the stratification of the survey design, weighted statistical methods were applied to produce nationally representative estimates. A sampling weight equal to the inverse probability of unit selection was allocated to each participant from the same stratum (defined with gender and age categories). This stratum sampling weight was defined as the ratio between the population stratum size and the observed sample stratum size.

Associations between the prevalence of vitamin D deficiency and insufficiency [serum 25(OH)D <25 nmol/L; <50 nmol/L, respectively] and a range of socio-demographic, behavioral, and clinical factors of interest were examined by using logistic regression. Vitamin D deficiency or insufficiency was considered as the dependent variable, while age, gender, education level, country of birth, smoking status, alcohol consumption, physical activity, dairy intake, abdominal obesity, global obesity, self-perception of health, and HDL-cholesterol level were the independent covariates. “Low risk” participants (younger age, women, Luxembourgish, living above poverty threshold, non-smokers, nondrinkers, physically active, excellent/good health perception, having no abdominal or global obesity, having high level of LDL-cholesterol, and increasing daily dairy intake) were considered as reference categories. Selection of variables in the multivariate logistic regression analyses was based on a literature review, scientific biological rationale and on statistical criteria (variables showing *p* < 0.05 in univariate models). Interactions between age (in years) and the others variables included in the multivariate model were all not significant and thus not included in the final model.

Several sensitivity analyses were performed to confirm the robustness of the presented findings. Results were considered significant at the 5% critical level (*p* < 0.05, 2 sided). All statistical analyses were performed using PASW^®^ for Windows^®^ version 21.0 software (formerly SPSS Statistics, Inc. Quarry Bay, Hong Kong) and survey procedure for complex sampling designs.

## 3. Results

### 3.1. Vitamin D Status

**Figure 1:**
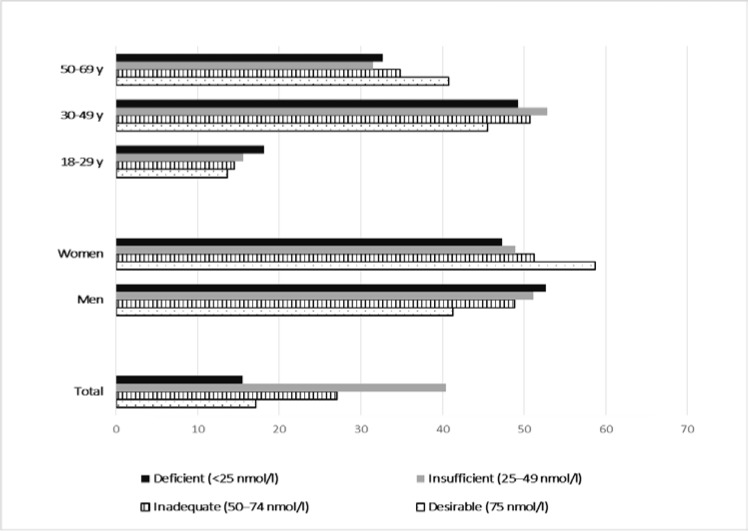
Total, sex- and age-specific prevalence of different categories of vitamin D, among the adults in Luxembourg, ORISCAV-LUX study.

Figure 1 depicts total, sex- and age-specific prevalence of different categories of vitamin D status. Overall, only 17.1% of the population had a “desirable” serum 25(OH)D level >75 nmol/L, whereas 27.1%, 40.4% and 15.5% had “inadequate”, “insufficient” and “deficient” levels, respectively. There was no age- or sex-specific difference in vitamin D status (*p* = 0.20, *p* = 0.053, respectively).

The mean (±SE) serum 25(OH)D concentration was 53.6 ± 0.7 nmol/L (median 49; Q1 = 36; Q3 = 68). Mean serum vitamin D levels were significantly higher in women than in men (56.6 ± 1.1 *vs.* 51.0 ± 0.9, *p* = 0.008, respectively). Participants of non-Europid origin and from Portugal had 25(OH)D levels 8–9 nmol/L lower than other Europeans and from Luxembourgish participants (*p* < 0.0001).

### 3.2. Prevalence of Vitamin D Deficiency and Insufficiency by Risk Factors

Table 1 presents the prevalence of vitamin D deficiency and insufficiency [serum 25(OH)D level <25 nmol/L; <50 nmol/L, respectively]. Concerning vitamin D deficiency, the prevalence was greater among current smokers compared to non-smokers (22.7% *vs.* 13.4%). Regardless of age and sex, current smokers were about two times as likely of having a vitamin D deficiency compared to non-smokers (age and sex-adjusted OR = 1.88; 95%CI 1.35–2.63; *p* = 0.0002). Likewise, abdominally obese subjects, and those having reduced HDL level were at higher odds of vitamin D deficiency than their counterparts (age and sex-adjusted OR = 1.44; 95%CI 1.05–1.99, *p* = 0.021; and OR = 1.54; 95%CI 1.05–2.26, *p* = 0.026, respectively). Interestingly, subjects who self-perceived their health as fair/poor had significantly higher odds of having vitamin D deficiency than those who self-rated their health as excellent/good, irrespective of their age and sex (age and sex-adjusted OR = 1.74; 95%CI 1.28–2.36, *p* = 0.0005).

Considering vitamin D insufficiency, in addition to the previous factors (smoking status, abdominal obesity, high HDL level and health perception), the prevalence was significantly different according to country of birth, alcohol consumption and obesity status. Participants from non-European countries were at higher odds compared to Luxembourgers (age and sex-adjusted OR = 1.65; 95%CI 1.002–2.74, *p* = 0.02), likewise abdominally obese compared to subjects with normal WC (age and sex-adjusted OR = 1.71; 95% CI 1.33–2.20, *p* > 0.0001). Irrespective of age and sex, alcohol drinkers were at lower odds of having vitamin D insufficiency than non-drinkers (age and sex-adjusted OR = 0.72; 95%CI 0.53–0.97, *p* = 0.03) (Table 1).

### 3.3. Independent Correlates to Vitamin D Deficiency and Insufficiency

Respectively, Table 2 and Table 3 present the vitamin D deficiency and insufficiency models from multivariate logistic regression analyses. In case of vitamin D deficiency, only smoking status, remained significantly and independently associated with vitamin D deficiency (*p* = 0.0012). Smoking increased two-fold the odd ratio of vitamin D deficiency as compared with non-smokers (fully adjusted OR = 1.80; 95%CI 1.26–2.57, *p* = 0.006) (Table 2).

In case of vitamin D insufficiency, increased age was significantly associated with lower odds (OR = 0.99; 95%CI 0.98–0.99, *p* = 0.023), whereas obesity, defined as BMI > 30 kg/m^2^, increased two-fold the odd ratio of vitamin D insufficiency (OR = 1.942; 95%CI 1.29–2.93) (Table 3).

**Table 1:** Prevalence and age- and sex-adjusted odds ratios (ORs) for vitamin D deficiency and insufficiency by risk factors, in adults’ participants to the ORISCAV-LUX study (*n* = 1335 subjects).

	Vitamin D Deficiency					Vitamin D Insufficiency				
Characteristics	Total Subjects	No. (%) of Subjects with Vitamin D Insufficiency *	Age and sex adjusted OR †	95%CI	*p-Value*	Total Subjects	No. (%) of Subjects with Vitamin D Insufficiency *	Age and Sex Adjusted OR †	95%CI	*p-Value*
Level of education					0.27					0.53
* Tertiary*	351	47 (13.4%)	Ref.			351	187 (53.3%)	Ref.		
* Secondary*	625	95 (15.2%)	1.41	0.92–2.14		625	347 (55.5%)	1.11	0.85–1.44	
* Primary*	345	60 (17.4%)	1.168	0.8–1.7		345	194 (56.2%)	1.18	0.87–1.6	
Country of birth					0.21					0.02
* Luxembourg*	813	126 (15.5%)	Ref			813	440 (54.1%)	Ref.		
* Other European country*	159	25 (15.7%)	0.99	0.62–1.6		288	145 (50.3%)	0.86	0.66–1.13	
* No European country*	288	37 (12.9%)	0.81	0.54–1.2		75	50 (66.7%)	1.65	1.002–2.74	
* Portugal*	75	17 (22.7%)	1.58	0.9–2.9		159	100 (62.9%)	1.38	0.97–1.97	
Economic status					0.58					0.26
* Above risk of poverty threshold*	907	138 (15.2%)	Ref.			907	494 (54.5%)	Ref.		
* Below risk of poverty threshold*	251	42 (16.7%)	1.11	0.76–1.63		251	148 (58.9%)	1.17	0.88–1.56	
Smoking status					0.0002					0.02
* Non–smokers*	1049	140 (13.4%)	Ref			1049	558 (53.2%)	Ref.		
* Current smokers*	286	65 (22.7%)	1.88	1.35–2.63		286	177 (38.1%)	1.37	1.044–1.79	
Alcohol consumption					0.81					0.03
* Non-drinker*	226	35 (15.5%)	Ref			226	137 (60.6%)	Ref.		
* Drinker*	1109	170 (15.3%)	0.95	0.63–1.42		1109	598 (53.92%)	0.72	0.53–0.97	
Physical activity					0.63					0.58
* Active*	1052	161 (15.3%)	Ref			1052	575 (54.7%)	Ref.		
* Inactive*	221	37 (16.7%)	1.1	0.74–1.62		221	126 (57%)	1.08	0.81–1.45	
Obesity status					0.02					<0.0001
* Underweight*	21	5 (23.8%)	1.93	0.66–5.61		21	13 (61.9%)	1.52	0.58–3.950	
* Normal weight*	566	77 (13.6%)	Ref			566	280 (49.5%)	Ref.		
* Overweight*	446	61 (13.7%)	1.02	0.69–1.48		446	239 (53.6%)	1.24	0.96–1.62	
* Obesity*	301	62 (20.6%)	1.72	1.16–2.55		301	202 (67.11%)	2.33	1.71–3.18	
Abdominal obesity §					0.021					<0.0001
* No Obese*	611	83 (13.6%)	Ref			611	315 (51.5%)	Ref.		
* Obese*	723	122 (16.9%)	1.44	1.05–1.99		723	419 (57.95%)	1.71	1.33–2.2	
HDL level £					0.026					0.001
* High HDL-C*	1064	150 (14.10%)	Ref			1064	563 (52.9%)	Ref.		
* Reduced HDL-C*	243	48 (19.75%)	1.54	1.05–2.26		243	154 (63.4%)	1.64	1.22–2.21	
Self–perceived health					0.0005					0.0049
* Excellent/good*	818	103 (12.6%)	Ref			818	425 (51.96%)	Ref.		
* Fair/Poor*	485	96 (19.8%)	1.74	1.28–2.36		485	289 (59.59%)	1.39	1.10–1.75	
Vitamin D intake, µg/day		9.3 [9.1–9.6]	0.51	0.29–0.87	0.01		2.6 [1.4–4.6]	0.97	0.93–1.009	0.12
Serum Ca, mg/dL		1.2 [0.7–2.05]	1.022	0.9–1.15	0.73		9.3 [9.1–9.6]	0.95	0.72–1.25	0.71
Dairy intake, servings/day		2.6 [1.4–4.6]	0.92	0.87–0.98	0.01		1.2 [0.7–2.05]	1.008	0.9–1.09	0.83

Ref., referent category. * Vitamin D deficiency defined as a 25(HD)D level of less than 25 nmol/L. Vitamin D insufficiency defined as a 25(HD)D level of less than 50 nmol/L. Data indicate Number (%), otherwise median [interquartile]; † OR adjusted for age and gender. § Abdominally obese subjects defined as WC ≥ 102 cm for men and ≥88 cm for women; £ Reduced concentration of HDL-C <40 mg/dL for men and <50 mg/dL for women.

**Table 2:** Independent demographic, socioeconomic, and behavioral correlates of vitamin D deficiency in adults’ participants to the ORISCAV-LUX study, as identified by multivariate logistic regression (*n* = 1277 subjects).

		Vitamin D Deficiency *		
Characteristics		Fully adjusted OR †	95%CI	*p*-value
Sex	*Men v. women*	1.29	0.9–1.8	0.13
Age, years		0.99	0.98–1.007	0.36
HDL level	*Low level v. high level*	1.24	0.83–1.84	0.29
Obesity status				0.16
	*Underweight v. normal weight*	1.43	0.45–4.54	
	*Overweight v. normal weight*	0.82	0.52–1.29	
	*Obesity v. normal weight*	1.32	0.76–2.27	
Abdominal obesity	*Abdominally obese v. no-abdominally obese*	1.28	0.81–2.03	0.28
Smoking status	*Smokers v. non-smokers*	1.80	1.26–2.57	0.0012
Health perception	*Fair/poor v. excellent/good health*	1.37	0.99–1.89	0.060

* Defined as a 25(HD) D level of less than 25 nmol/L. † OR adjusted for other demographic, socio-economic, dietary and lifestyle factors.

In case of vitamin D insufficiency, increased age was significantly associated with lower odds (OR = 0.99; 95%CI 0.98–0.99, *p* = 0.023), whereas obesity, defined as BMI > 30 kg/m^2^, increased two-fold the odd ratio of vitamin D insufficiency (fully adjusted OR = 1.94; 95%CI 1.29–2.93). After full adjustment, the association between alcohol consumption and vitamin D insufficiency became non-significant (Table 3).

**Table 3:** Independent demographic, socioeconomic, and behavioral correlates of vitamin D insufficiency in adults’ participants to the ORISCAV-LUX study, as identified by multivariate logistic regression (*n* = 1277 subjects).

		Vitamin D Insufficiency *		
Characteristics		Fully adjusted OR †	95% CI	*p*-value
Sex	*Men v. women*	1.25	0.97–1.60	0.081
Age, years		0.99	0.98–0.99	0.023
HDL level	*Low level v. high level*			0.083
Country of birth	*Other European country v. Luxembourg*	0.87	0.66–1.16	0.07
	*Non–European country v. Luxembourg*	1.70	1.009–2.87	
	*Portugal v. Luxembourg*	1.26	0.87–1.84	
Obesity status				0.0046
	*Underweight v. normal weight*	1.43	0.56–3.63	
	*Obesity v. normal weight*	1.94	1.29–2.93	
	*Overweight v. normal weight*	1.11	0.81–1.53	
Alcohol consumption	*Drinker v. non-drinker*	0.79	0.58–1.09	0.16
Abdominal obesity	*Abdominally obese v. no-abdominally obese*	1.11	0.81–1.52	0.53
Smoking status	*Smokers v. non-smokers*	1.28	0.96–1.71	0.093
Health perception	*Fair/poor v. excellent/good health*	1.08	0.84–1.38	0.54

* Defined as a 25(HD) D level of less than 50 nmol/L. † OR adjusted for other demographic, socio-economic, dietary and lifestyle factors.

## 4. Discussion

Vitamin D deficiency and insufficiency have been documented as a frequent public health problem in Europe and worldwide. This is the first nationwide study to describe the epidemiology of vitamin D status by comparing the prevalence of vitamin D deficiency and insufficiency by a range of socio-demographic, behavioral, and clinical characteristics among healthy adults living in Luxembourg.

Our findings indicate that vitamin D status in Luxembourg is alarming, as more than 80% of adults have inadequate levels of vitamin D [25 (OH)D less than 75 nmol/L], including 40% of people with insufficient level (<50 nmo/L) and 15% with an overt deficiency of less than 25 nmol/L.

Serum 25(OH)D concentrations lower than 25 nmol/L has been reported in 2%–30% of European adult populations, particularly in some ethnic groups and up to 80% in older institutionalized subjects [15,26]. The great variations in published data make direct international comparisons difficult, not only because of important methodological differences with respect to the characteristics of the target population, the study design, the sample selection, and the year of conduct, but also due to the variety of cut-off points being used in European population studies [25]. Nevertheless, our national population-based data are in line with previous studies and provide compelling evidence that inadequate vitamin D status is a prevailing and neglected public health problem in Luxembourg, particularly among smokers and obese subjects.

Despite emergent research on vitamin D status and its implications in a broad range of skeletal and non-skeletal diseases, the blood levels of 25(OH) D that define vitamin D deficiency or insufficiency remain a matter of debate. There is no international agreement as to what the optimal serum 25(OH)D concentration should be for human health. While there is a general European consensus that blood 25(OH)D levels below 25 nmol/L (or 10 ng/mL) qualify as “deficient” [3], recent American guidelines suggest however that concentrations <50 and <75 nmol/L represent vitamin D deficiency and insufficiency, respectively [27]. Additionally, the American Institute of Medicine have recently stated that a level of 50 nmol/L meets the needs of 97.5% of the population across all life-stage groups, and the IOM report classifies levels <30 nmol/L as deficient [2]. Indeed, studies in North America used significantly higher values to define inadequate vitamin D status than in Europe, which could be explained by the routine fortification of several foods in the US (e.g., milk) [3]. In the present study, we used the European normative values to define vitamin D status and deficiency, as low vitamin D status, particularly at levels below 25 nmol/L, is well recognized to have clinically adverse effects on musculoskeletal health in adults, including osteomalacia, proximal myopathy, secondary hyperparathyroidism and osteoporosis [13,27]. It should be stressed that vitamin D deficiency is easily treatable, so high prevalence of vitamin D deficiency or insufficiency is unacceptable in our country.

There are a number of potential factors that may have contributed to the relatively high prevalence of vitamin D deficiency and insufficiency among adults in Luxembourg. Compared to normal weight subjects, the prevalence of vitamin D deficiency [serum 25(OH)D level <25 nmol/L] was greater among underweight and obese subjects, and they were significantly at higher odds, after adjustment for age and sex. Likewise, abdominally obese subjects were at higher odds for vitamin D deficiency than non-abdominally obese subjects. However, these associations became non-significant after adjustment for other confounding factors, in the multivariate model. Adiposity is a well-known risk factor for vitamin D deficiency [28,29]. Although the explanation for the increased risk of vitamin D deficiency in obesity is unclear, it has been postulated that obese individuals tend to be less active and may avoid exposure to solar ultraviolet (UV) radiation, which is indispensable for the cutaneous synthesis of vitamin D3 [30]. Alternatively, it has been suggested that the metabolic clearance of vitamin D may increase in obesity, possibly with enhanced uptake by adipose tissue [31].

Regarding lifestyle characteristics, univariate and multivariate analysis demonstrated that the odds for vitamin D deficiency increased significantly and independently among smokers compared to non-smokers. Smokers were at two-fold odds to have deficiency compared to non-smokers. These findings are consistent with previous studies [32,33]. Literature has shown that an increased bone loss was registered in smokers [34], and tobacco smoking was associated with a low bone mass and an increased risk of osteoporotic fracture [35]. Brot *et al.* demonstrated that smoking has a significant effect on calcium and vitamin D metabolism, irrespective to confounding lifestyle factors [32]. Several hypotheses have been suggested concerning the mechanisms by which smoking affects vitamin D metabolism and hence bone health; the main focus being on the anti-estrogenic effect [32]. Smokers have reduced levels of circulating estrogens due to an increased hepatic turnover [36], resulting hence in an increased early bone loss [32]. Other lifestyle factors are more prevalent among smokers compared to nonsmokers such as less outdoor physical activity [37], and less compliance to dietary recommendations [38], with related nutritional deficiencies, all of which might play a role. Additionally, a direct toxic effect of tobacco smoking on bone cells is also a possibility [39].

Although the mechanisms driving the association are still unknown, epidemiologic evidence has shown there is an inverse relationship between circulating levels of 25(OH)D and cardiometabolic-risk biomarkers [40], including lipid biomarkers [41,42]. Numerous studies have shown that high serum concentrations of 25(OH)D are associated with favorable lipid profiles [43]. Consistently, our study showed an association between an increased risk of low 25(OH)D levels in participants with low HDL-C levels (F < 50, M < 40 mg/dL). This association did, however, vanish after further adjustment for demographic, socio-economic, dietary and lifestyle factors. Indeed, we cannot rule out that the positive associations between vitamin D and some lipid parameters may be biased by outdoor activities. From a public health standpoint, these findings are of significance, as they stress the need to promote sensible and moderate sun exposure, which represents the most important natural source of vitamin D [44,45]. Although vitamin D can be supplemented, the most cost-effective prevention strategy should focus on regular weekly UVB exposure, rather than supplement use/food fortification to improve vitamin D status. Additionally, the impact of correcting vitamin D deficiency on blood lipids (strong cardiovascular disease prognostic factors) has not yet been established. To date, it is still unknown whether low vitamin D levels cause cardiovascular disease or whether vitamin D status is simply a marker of health [46]. This is relevant for practitioners and the general population because of the increasing consumption of pharmacological doses of vitamin D sold over the counter [43]. The association of vitamin D deficiency with an unfavorable lipid profile has not been replicated in prospective studies. A recent longitudinal analysis showed that increasing 25(OH)D levels from the deficient to the optimal range (repletion group) compared with remaining in the deficient range (control group) was associated with small and clinically minimal effects on total cholesterol (0.8-mg/dL increase) and HDL cholesterol (0.4-mg/dL increase) and no significant changes in LDL cholesterol or triglycerides levels [43]. Failure to demonstrate significant improvements in these lipid parameters, suggests that correcting vitamin D deficiency with dietary supplements might not translate into clinically meaningful changes in lipid concentrations [43]. Data from intervention trials are required to confirm these findings. Kazlauskaite and colleagues [47] suggested that vitamin D may protect against cardiovascular risk by promoting formation of large HDL_2_ particles, affecting reverse cholesterol transport, which is an important atheroprotective HDL particle function [48]. However, epidemiological data are conflicting as other studies have suggested that higher small dense HDL_3_-C levels are most strongly associated with lower risk [49,50,51]. Most recent research indicated that low HDL_3_, but not HDL_2_ or HDL cholesterol is associated with an increased risk for long-term hard clinical events [52]. These studies varied in design, adjustment for confounders, and methods for HDL subclass separation and quantification. Unfortunately, data on HDL-C sub classification were not available in the present study.

Our findings indicated a statistical borderline relationship (*p* = 0.061) with self-perceived health, suggesting that vitamin D deficiency may also represent a marker of poor general wellbeing, that is, people with undetected disease conditions may experience lower vitamin D status as a consequence of their poor health.

Several strong points characterize the study. First, the recruitment of a large nationwide, population-based, representative sample of apparently healthy adults in Luxembourg. Second, a detailed study of non-participants showed that the demographic and clinical characteristics of the ORISCAV-LUX participants were comparable with those of non-participants, hence ruling out the possibility of selection bias in our study population [19]. Third, the data were weighted to provide population-representative prevalence estimates. Fourth, the assessment of 25 (OH) D was done in one central laboratory using the same methodology. Additionally, the ORISCAV-LUX measured a large set of potential related risk factors that have been rarely investigated in similar studies. We trust that our findings contribute to filling gaps on the worldwide map examining heterogeneities in vitamin D status [53].

However, similar to most of the population-based studies, the ORISCAV-LUX survey has some limitations, related mainly to the cross-sectional design which precluded any conclusion on causal relationship between vitamin D deficiency or insufficiency and the identified correlated factors. Second, no information was available on sun exposure practice, including time spent outdoors or sunscreen use. Consistent with most previous studies, there was a significant seasonal variation in 25-hydroxyvitamin D level. However, recruitment was conducted over a period of 15 months, between November 2007 and January 2009, which allowed balance in the effect of seasonal variability. Additionally, there were no changes in the current findings and the final conclusion after performing a sensitivity analysis by introducing this variable in the multivariable model (data not shown in this manuscript).

## 5. Conclusions

Our data demonstrates a high prevalence of vitamin D deficiency and insufficiency in otherwise healthy adults in Luxembourg. The prevalence was particularly high among young adults, smokers and obese subjects. Along with the effect of vitamin D deficiency and insufficiency on the risk of several diseases, cancer and mortality, our findings have practical implications for public health dietary recommendations. Vitamin D deficiency can be effectively treated through oral repletion. Vitamin D fortification and supplementation may represent a cost-effective public health strategy to tackle the deficiency and to maintain healthy bone density, lower risk for fractures and improve global human health. However, data from intervention trials are required to confirm the purported benefits of vitamin D repletion on the lipid profile inferred from cross-sectional studies [43,46]. With increasing consumption of pharmacological doses of vitamin D sold over the counter, our findings are of particular importance for healthcare practitioners and policy makers. Vitamin D deficiency represents the tip of the iceberg of inappropriate vitamin D status. From a public health perspective, this study provides an overview of vitamin D status in Luxembourg, and a basis for evaluating whether measures for improving deficiency and insufficiency should be taken.

## References

[1] Palacios C. (2006). The role of nutrients in bone health, from A to Z. Crit. Rev. Food Sci. Nutr..

[2] Ross A.C., Manson J.E., Abrams S.A., Aloia J.F., Brannon P.M., Clinton S.K., Durazo-Arvizu R.A., Gallagher J.C., Gallo R.L., Jones G. (2011). The 2011 report on dietary reference intakes for calcium and vitamin D from the Institute of Medicine: What clinicians need to know. J. Clin. Endocrinol. Metab..

[3] Spiro A., Buttriss J.L. (2014). Vitamin D: An overview of vitamin D status and intake in Europe. Nutr. Bull. BNF.

[4] Dunnigan M.G., Henderson J.B., Hole D. (1990). Serum 25-hydroxyvitamin D and colon cancer. Lancet.

[5] Garland C.F., Garland F.C., Gorham E.D., Lipkin M., Newmark H., Mohr S.B., Holick M.F. (2006). The role of vitamin D in cancer prevention. Am. J. Public Health.

[6] Cannell J.J., Vieth R., Umhau J.C., Holick M.F., Grant W.B., Madronich S., Garland C.F., Giovannucci E. (2006). Epidemic influenza and vitamin D. Epidemiol. Infect..

[7] Laaksi I., Ruohola J.P., Tuohimaa P., Auvinen A., Haataja R., Pihlajamaki H., Ylikomi T. (2007). An association of serum vitamin D concentrations <40 nmol/L with acute respiratory tract infection in young Finnish men. Am. J. Clin. Nutr..

[8] Munger K.L., Levin L.I., Hollis B.W., Howard N.S., Ascherio A. (2006). Serum 25-hydroxyvitamin D levels and risk of multiple sclerosis. JAMA.

[9] Poole K.E., Loveridge N., Barker P.J., Halsall D.J., Rose C., Reeve J., Warburton E.A. (2006). Reduced vitamin D in acute stroke. Stroke.

[10] Autier P., Boniol M., Mullie P. (2014). Vitamin D status and ill health: A systematic review. Lancet Diabetes Endocrinol..

[11] Autier P., Gandini S. (2007). Vitamin D supplementation and total mortality: A meta-analysis of randomized controlled trials. Arch. Intern. Med..

[12] Lappe J.M., Travers-Gustafson D., Davies K.M., Recker R.R., Heaney R.P. (2007). Vitamin D and calcium supplementation reduces cancer risk: Results of a randomized trial. Am. J. Clin. Nutr..

[13] Lips P. (2001). Vitamin D deficiency and secondary hyperparathyroidism in the elderly: Consequences for bone loss and fractures and therapeutic implications. Endocr. Rev..

[14] Daly R.M., Gagnon C., Lu Z.X., Magliano D.J., Dunstan D.W., Sikaris K.A., Zimmet P.Z., Ebeling P.R., Shaw J.E. (2012). Prevalence of vitamin D deficiency and its determinants in Australian adults aged 25 years and older: A national, population-based study. Clin. Endocrinol..

[15] Lips P. (2010). Worldwide status of vitamin D nutrition. J. Steroid Biochem. Mol. Biol..

[16] Holick M.F. (2007). Vitamin D deficiency. N. Engl. J. Med..

[17] Gordon C.M., de Peter K.C., Feldman H.A., Grace E., Emans S.J. (2004). Prevalence of vitamin D deficiency among healthy adolescents. Arch. Pediatr. Adolesc. Med..

[18] Alkerwi A., Sauvageot N., Donneau A.F., Lair M.L., Couffignal S., Beissel J., Delagardelle C., Wagener Y., Albert A., Guillaume M. (2010). First nationwide survey on cardiovascular risk factors in Grand-Duchy of Luxembourg (ORISCAV-LUX). BMC Public Health.

[19] Alkerwi A., Sauvageot N., Couffignal S., Albert A., Lair M.L., Guillaume M. (2010). Comparison of participants and non-participants to the ORISCAV-LUX population-based study on cardiovascular risk factors in Luxembourg. BMC Med. Res. Methodol..

[20] Alkerwi A., Donneau A.F., Sauvageot N., Lair M.L., Albert A., Guillaume M. (2012). Dietary, behavioural and socio-economic determinants of the metabolic syndrome among adults in Luxembourg: Findings from the ORISCAV-LUX study. Public Health Nutr..

[21] Committee I.R. Guidelines for Data Processing and Analysis of the International Physical Activity Questionnaire (IPAQ)—Short and Long Forms. http://wwwipaqkise/scoringpdf.

[22] Alberti K.G., Zimmet P., Shaw J., Group IDFETFC (2005). The metabolic syndrome—A new worldwide definition. Lancet.

[23] Grundy S.M., Brewer H.B., Cleeman J.I., Smith S.C., Lenfant C., National Heart, Lung, and Blood Institute, American Heart Association (2004). Definition of metabolic syndrome: Report of the National Heart, Lung, and Blood Institute/American Heart Association conference on scientific issues related to definition. Circulation.

[24] Sauvageot N., Alkerwi A., Albert A., Guillaume M. (2013). Use of food frequency questionnaire to assess relationships between dietary habits and cardiovascular risk factors in NESCAV study: Validation with biomarkers. Nutr. J..

[25] Hilger J., Friedel A., Herr R., Rausch T., Roos F., Wahl D.A., Pierroz D.D., Weber P., Hoffmann K. (2014). A systematic review of vitamin D status in populations worldwide. Br. J. Nutr..

[26] Mithal A., Wahl D.A., Bonjour J.P., Burckhardt P., Dawson-Hughes B., Eisman J.A., El-Hajj Fuleihan G., Josse R.G., Lips P., Morales-Torres J. (2009). Global vitamin D status and determinants of hypovitaminosis D. Osteoporos. Int..

[27] Holick M.F., Binkley N.C., Bischoff-Ferrari H.A., Gordon C.M., Hanley D.A., Heaney R.P., Murad M.H., Weaver C.M., Endocrine Society (2011). Evaluation, treatment, and prevention of vitamin D deficiency: An Endocrine Society clinical practice guideline. J. Clin. Endocrinol. Metab..

[28] Wortsman J., Matsuoka L.Y., Chen T.C., Lu Z., Holick M.F. (2000). Decreased bioavailability of vitamin D in obesity. Am. J. Clin. Nutr..

[29] Forsythe L.K., Livingstone M.B., Barnes M.S., Horigan G., McSorley E.M., Bonham M.P., Magee P.J., Hill T.R., Lucey A.J., Cashman K.D. (2012). Effect of adiposity on vitamin D status and the 25-hydroxycholecalciferol response to supplementation in healthy young and older Irish adults. Br. J. Nutr..

[30] Compston J.E., Vedi S., Ledger J.E., Webb A., Gazet J.C., Pilkington T.R. (1981). Vitamin D status and bone histomorphometry in gross obesity. Am. J. Clin. Nutr..

[31] Liel Y., Ulmer E., Shary J., Hollis B.W., Bell N.H. (1988). Low circulating vitamin D in obesity. Calcif. Tissue Int..

[32] Brot C., Jorgensen N.R., Sorensen O.H. (1999). The influence of smoking on vitamin D status and calcium metabolism. Eur. J. Clin. Nutr..

[33] Cutillas-Marco E., Fuertes-Prosper A., Grant W.B., Morales-Suarez-Varela M. (2012). Vitamin D deficiency in South Europe: Effect of smoking and aging. Photodermatol. Photoimmunol. Photomed..

[34] Vogel J.M., Davis J.W., Nomura A., Wasnich R.D., Ross P.D. (1997). The effects of smoking on bone mass and the rates of bone loss among elderly Japanese-American men. J. Bone Miner. Res..

[35] Law M.R., Hackshaw A.K. (1997). A meta-analysis of cigarette smoking, bone mineral density and risk of hip fracture: Recognition of a major effect. BMJ.

[36] Daniel M., Martin A.D., Drinkwater D.T. (1992). Cigarette smoking, steroid hormones, and bone mineral density in young women. Calcif. Tissue Int..

[37] Thorlindsson T., Vilhjalmsson R. (1991). Factors related to cigarette smoking and alcohol use among adolescents. Adolescence.

[38] Alkerwi A., Sauvageot N., Nau A., Lair M.L., Donneau A.F., Albert A., Guillaume M. (2012). Population compliance with national dietary recommendations and its determinants: Findings from the ORISCAV-LUX study. Br. J. Nutr..

[39] Fang M.A., Frost P.J., Iida-Klein A., Hahn T.J. (1991). Effects of nicotine on cellular Function in UMR 106-01 osteoblast-like cells. Bone.

[40] Gannage-Yared M.H., Chedid R., Khalife S., Azzi E., Zoghbi F., Halaby G. (2009). Vitamin D in relation to metabolic risk factors, insulin sensitivity and adiponectin in a young Middle-Eastern population. Eur. J. Endocrinol. Eur. Fed. Endocr. Soc..

[41] Jorde R., Grimnes G. (2011). Vitamin D and metabolic health with special reference to the effect of vitamin D on serum lipids. Prog. Lipid Res..

[42] Jorde R., Figenschau Y., Hutchinson M., Emaus N., Grimnes G. (2010). High serum 25-hydroxyvitamin D concentrations are associated with a favorable serum lipid profile. Eur. J. Clin. Nutr..

[43] Ponda M.P., Huang X., Odeh M.A., Breslow J.L., Kaufman H.W. (2012). Vitamin D may not improve lipid levels: A serial clinical laboratory data study. Circulation.

[44] Zittermann A. (2006). Vitamin D and disease prevention with special reference to cardiovascular disease. Prog. Biophys. Mol. Biol..

[45] Zittermann A. (2014). Vitamin D and cardiovascular disease. Anticancer Res..

[46] Rosen C.J. (2011). Clinical practice. Vitamin D insufficiency. N. Engl. J. Med..

[47] Kazlauskaite R., Powell L.H., Mandapakala C., Cursio J.F., Avery E.F., Calvin J. (2010). Vitamin D is associated with atheroprotective high-density lipoprotein profile in postmenopausal women. J. Clin. Lipidol..

[48] Rye K.A., Bursill C.A., Lambert G., Tabet F., Barter P.J. (2009). The metabolism and anti-atherogenic properties of HDL. J. Lipid Res..

[49] Sweetnam P.M., Bolton C.H., Yarnell J.W., Bainton D., Baker I.A., Elwood P.C., Miller N.E. (1994). Associations of the HDL2 and HDL3 cholesterol subfractions with the development of ischemic heart disease in British men. The Caerphilly and Speedwell Collaborative Heart Disease Studies. Circulation.

[50] Yu S., Yarnell J.W., Sweetnam P., Bolton C.H. (2003). High density lipoprotein subfractions and the risk of coronary heart disease: 9-years follow-up in the Caerphilly Study. Atherosclerosis.

[51] Parish S., Offer A., Clarke R., Hopewell J.C., Hill M.R., Otvos J.D., Armitage J., Collins R., Heart Protection Study Collaborative Group (2012). Lipids and lipoproteins and risk of different vascular events in the MRC/BHF Heart Protection Study. Circulation.

[52] Martin S.S., Khokhar A.A., May H.T., Kulkarni K.R., Blaha M.J., Joshi P.H., Toth P.P., Muhlestein J.B., Anderson J.L., Knight S. (2015). HDL cholesterol subclasses, myocardial infarction, and mortality in secondary prevention: The Lipoprotein Investigators Collaborative. Eur. Heart J..

[53] Wahl D.A., Cooper C., Ebeling P.R., Eggersdorfer M., Hilger J., Hoffmann K., Josse R., Kanism J.A., Mithal A., Pierroz D.D. (2012). A global representation of vitamin D status in healthy populations. Arch. Osteoporos..

